# From Alginate to Pixel: Comparing the Effect of Two Dental Impression Methods on Children’s Anxiety

**DOI:** 10.3390/children12070866

**Published:** 2025-06-30

**Authors:** Isabel Cristina Miranda Ataíde, Clara Serna-Muñoz, Cristina Maria Ferreira Guimaraes Pereira Areias, Álvaro Amadeu Ferreira de Azevedo, Romeu Eduardo Pereirinha Henriques Ferreira de Andrade, Antonio José Ortiz-Ruiz

**Affiliations:** 1 Faculty of Dental Medicine, University of Porto, 4200-393 Porto, Portugal; isabel.ataide.mdentaria@gmail.com (I.C.M.A.); romeupandrade@gmail.com (R.E.P.H.F.d.A.); 2 Faculty of Medicine, University of Murcia, 30120 Murcia, Spain; 3 Children’s Integrated Dental Clinic, Department of Dermatology, Stomatology, Radiology and Physical Medicine, University of Murcia, 30008 Murcia, Spain; 4 Biomedical Research Institute of Murcia Pascual Parrilla-IMIB, 30120 Murcia, Spain; ajortiz@um.es; 5 RISE-Health, I&D Unit, Faculty of Dental Medicine, Oporto University, 4200-393 Porto, Portugal; careias@fmd.up.pt (C.M.F.G.P.A.); aazevedo@fmd.up.pt (Á.A.F.d.A.); 6 Department of Integrated Pediatric Dentistry, Faculty of Medicine, University of Murcia, 30008 Murcia, Spain

**Keywords:** alginate impression, analogic impression, children, dental fear/anxiety/stress, heart rate, intraoral scanner, oxygen saturation

## Abstract

Background/Objectives: Alginate dental impressions are often among the most anxiety-inducing procedures for pediatric patients due to discomfort caused by nausea, gagging, and the vomiting reflex. This technique frequently raises anxiety levels in children. In contrast, intraoral scanners are increasingly used in clinical practice and offer a more comfortable alternative. Physiological parameters such as heart rate (HR) and oxygen saturation (SpO_2_) are useful, objective indicators of anxiety. This study aimed to evaluate significant changes in HR and SpO_2_ during dental impression procedures using two techniques—an analog (alginate) and a digital (intraoral scanner) one—in both dental arches, to determine the method inducing the least anxiety. Methods: A non-interventional clinical study was conducted on a sample of 30 children. A fingertip pulse oximeter was used to measure HR and SpO_2_ during impression taking with alginate and with an intraoral scanner. Statistical analysis was performed using SPSS^®^ (Version 30.0. Amonk, NY, USA: IBM Corp). Descriptive statistics (mean and standard deviation) were calculated, and the Friedman and Wilcoxon tests with Bonferroni correction were applied, using a 5% significance level. The study was approved by the Ethics Committee of the University of Murcia. Results: Alginate impressions produced a statistically significant increase in HR in both the upper [(19 ± 11) bpm] and lower [(18 ± 9) bpm] arches compared to the scanner [(7 ± 5) bpm and (7 ± 4) bpm, respectively] (χ^2^ = 49.30; *p* < 0.001). SpO_2_ levels also dropped more when using alginate for both the upper and lower arches [(−2 ± 2)% and (−2 ± 3)%, respectively] than when using the scanner [(−1 ± 1)% in both arches] (χ^2^ = 21.41; *p* < 0.001). Conclusions: Alginate impressions triggered a significant anxiety response, as evidenced by significant changes in HR and SpO_2_. Intraoral scanners were less invasive, as indicated by the greater proximity of the physiological parameters to the baseline values, making them a promising alternative in pediatric dentistry.

## 1. Introduction

Anxiety and fear are frequently observed emotional responses in pediatric dental procedures [1,2]. In the context of dentistry, anxiety is defined as a negative emotional state characterized by apprehension and often accompanied by a perceived loss of control in response to dental procedures [3,4,5,6]. Despite significant advancements in dental techniques, technologies, and materials, fear and anxiety associated with the dental setting and specific procedures remain a frequent reality among children worldwide [1,7,8].

Stress and anxiety influence a child’s ability to cope with dental treatment and can pose a significant challenge to the attending dentist [2,9,10,11]. Pediatric patients with anxiety during dental procedures may demonstrate reduced cooperation, require longer appointments, and exhibit disruptive behavior, leading to a taxing experience for both the children and the dental professionals involved [8,12].

Clinical success in pediatric dentistry not only depends on the technical skills of the practitioner, but also on the patients’ behavioral responses [13]. Emotional barriers such as fear and anxiety may affect treatment adherence and contribute to the early discontinuation of oral health care, a behavior that can persist into adulthood [2,10,14]. A 2024 systematic review estimated that approximately 30% of preschool-aged children experience fear of attending pediatric dental appointments [5].

Several methods have been used to assess dental anxiety and fear, including psychometric scales, questionnaires, and physiological indicators [5,6,10,15]. Among these, physiological parameters such as heart rate (HR) and blood oxygen saturation (SpO_2_) stand out as objective, non-invasive markers that are sensitive to stress-related physiological responses [1,2]. Negative emotions stimulate the release of endogenous catecholamines, resulting in an increased HR and changes in other physiological parameters [16,17]. Consequently, HR and SpO_2_ have been widely recognized as relevant and objective indicators in the assessment of pediatric anxiety [1,18,19]. Studies have demonstrated a positive correlation between changes in these physiological biomarkers and anxiety in the dental setting [1,20].

Procedures perceived as painful or uncomfortable—such as traditional dental impressions, especially those involving alginate—often trigger fear and anxiety during pediatric dental visits [3,21,22]. Alterations in an individual’s emotional state may lead to reduced pain tolerance, further intensifying the experience of anxiety [11,12]. Alginate impressions are among the most feared procedures in pediatric dentistry [23]. Although the analog technique is widely used due to its accessibility and reliability, it is often associated with sensations such as choking, nausea, and the activation of the gag reflex. These experiences can increase anxiety levels and contribute to the development of negative perceptions toward dental care among children [21,22,24]. Such phenomena during dental impressions may lead to the creation of long-term associations between dental visits and negative emotional responses, such as fear [11,17,21]. The occurrence of gag reflexes and nausea during alginate impressions represents a significant limitation in pediatric dentistry, highlighting the need for alternative strategies that minimize anxiety-inducing stimuli [2,9,24].

In this context, intraoral scanners have been gaining prominence in clinical dental practice due to the increased comfort they provide to patients during the impression process [22,25]. The use of intraoral scanners for producing study casts has seen substantial growth among dental practitioners worldwide [26,27]. Studies indicate that the digital technique provides high accuracy, shortens chair time, and reduces patient discomfort when compared to the conventional alginate technique [28,29,30].

Numerous studies have compared preference, comfort, and satisfaction between analog and digital impression techniques across various age groups. Digital impressions have been associated with greater comfort and consequently lower anxiety levels [22,25,26,28,29,31,32]. However, the use of HR and SpO_2_ as biological markers of anxiety during digital and analog impression making in children remains understudied. This study aims to contribute to a deeper understanding of the procedures that negatively influence children’s emotional experience during pediatric dental visits.

The primary objective of this study was to evaluate and compare the significant changes in HR and SpO_2_ during dental impression procedures using two different techniques—an analog (alginate) and a digital (intraoral scanner) one—on both dental arches, aiming to obtain conclusions that can help identifying the less anxiety-inducing method. The analysis of these physiological markers and their association with anxiety levels in the context of pediatric dentistry may support the adoption of clinical practices that reduce anxiety, enhance the patient–provider relationship, and improve treatment outcomes.

This investigation seeks to demonstrate the following hypotheses:

1. Higher levels of anxiety, reflected by greater increases in HR and more significant decreases in SpO_2_, will be detected during alginate impressions.
2. The use of intraoral scanning will result in greater stability in HR and SpO_2_.
3. From a physiological standpoint, intraoral scanning will be the less anxiety-inducing technique.

## 2. Materials and Methods

This non-interventional clinical and cross-sectional study was conducted from January to April 2025.

### 2.1. Study Sample and Setting

The sample comprised 30 healthy children aged between 5 and 11 years who were receiving dental care at the University Dental Clinic of the University of Murcia, Spain. Inclusion criteria encompassed patients within the specified age range who required dental impressions as part of their treatment plan, particularly for orthodontic purposes, such as space maintainers, and without any relevant medical history that could interfere with the physiological parameters assessed. Exclusion criteria included children with a prior diagnosis of behavioral disorders, generalized anxiety disorders, or any known systemic condition that could directly influence heart rate (HR) and/or oxygen saturation (SpO_2_). A total of 120 dental impressions were performed on the 30 children, with each participant undergoing both analog and digital impression making of the upper and lower dental arches.

To estimate the required sample size, preliminary data from the first 10 participants were analyzed, given the absence of prior studies with comparable methodology in the scientific literature from which to extract relevant parameters. Among the two primary outcomes considered, the change in oxygen saturation was selected as the basis for calculation, since heart rate change exhibited was high, which would have yielded an unrealistically small sample size.

Assuming a two-tailed test with a significance level (α) of 0.05 and a statistical power of 80% (1 − β = 0.80), it was determined that 28 participants per group would be required to detect a minimum clinically relevant difference of 0.6 units. This calculation is based on an estimated common standard deviation of 0.56 and an assumed correlation coefficient of 0.1 between baseline and post-intervention measurements. A dropout rate of 10% was incorporated into the final sample size estimation.

### 2.2. Ethical Considerations

The study was conducted in accordance with the ethical principles outlined in the Declaration of Helsinki. Ethical approval was granted by the Ethics Committee of the University of Murcia, under protocol number M10/2024/469, and the Data Protection Committee.

Bioethical principles pertinent to such investigations were strictly adhered to. Participant anonymity and data confidentiality were ensured through the assignment of unique codes to each participant. Confidentiality throughout data storage and processing was also ensured during all phases of the research and scientific dissemination.

Participants and their parents or legal guardians were thoroughly informed about the study’s objectives, procedures, and benefits, both verbally and via a written document. Participation proceeded only after obtaining informed consent. It was emphasized that participation was entirely voluntary and that the decision to participate or not would not affect the treatment provided at the university clinic. Freedom to authorize participation and the right to withdraw consent at any time without repercussions were guaranteed.

### 2.3. Data Collection

Continuous monitoring of SpO_2_ and HR during data collection was performed using the portable pediatric finger oximeter *KidsO2 Ring* (Wellue^®^, Shenzhen, China), a CE-certified medical device. Designed specifically for children, this device features a hypoallergenic silicone ring that adapts easily to various finger sizes.

Data management was facilitated via a smartphone using the *ViHealth* application, which allows real-time visualization of data and access to detailed, interactive measurement reports. The application provides statistics such as average, minimum, and maximum values for HR and SpO_2_. Reports can be exported in PDF or CSV formats, aiding in data analysis. The combination of this oximeter and the *ViHealth* application provided an ideal, non-invasive tool for physiological monitoring during the impression procedures.

### 2.4. Study Protocol

Participants underwent a standardized procedure protocol for dental impressions. The *KidsO2 Ring* oximeter was used to measure HR and SpO_2_. The protocol included an initial rest phase, two procedural phases (alginate and scanner), and an interval phase, all conducted on the same day by a single operator.

#### 2.4.1. Baseline Phase

In the initial phase (baseline phase), with the participant seated in the dental chair, HR and SpO_2_ were recorded using the finger oximeter. During this phase, participants were encouraged to interact with their parents or engage in simple activities, like drawing, to promote relaxation and obtain baseline physiological values. The oximeter remained connected for 2 to 5 min, during which HR and SpO_2_ values were continuously recorded and automatically stored in the *ViHealth* application for subsequent analysis.

#### 2.4.2. Oral Cavity Impression Phase

In this phase, each child underwent both digital and conventional impression techniques for both dental arches. The digital impressions were performed using the Primescan 3D^®^ intraoral scanner (Dentsply Sirona Inc., Charlotte, NC, USA), while the conventional impressions utilized alginate.

The sequence of techniques was randomly determined using *Microsoft*^®^ *Excel*^®^ (Version 16.62) to minimize bias from the selection order and accumulated fatigue. For categorization purposes, the following coding was used: “1 = Alginate, 2 = Digital.”

The finger oximeter was placed at the beginning of each impression and removed at the end of the procedure. After each measurement, relevant information was manually recorded in the *ViHealth* application notes, including the participant’s identification number, the type of technique used (alginate or scanner), and the corresponding dental arch (upper or lower).

Each participant had a total of five distinct HR and SpO_2_ recordings: baseline values obtained during the baseline phase; alginate impression—upper arch; alginate impression—lower arch; scanner impression—upper arch; and scanner impression—lower arch.

#### 2.4.3. Conventional Technique (Alginate)

The conventional technique was performed using *Zhermack Orthoprint*™ alginate (Dentsply Sirona Inc., Charlotte, NC, USA) and pediatric steel impression trays appropriate for each participant’s dental arch size, following the manufacturer’s instructions. The procedure was conducted with the participant seated, with the chair positioned at approximately 90°, ensuring stability and comfort. Prior to the procedure, the participant was informed about the alginate impression technique. HR and SpO_2_ were continuously monitored using the pediatric oximeter during the procedure, and data were stored in the *ViHealth* application.

The procedural steps included: pre-procedure explanation of the oral impression process; placement of the oximeter on the participant’s right index finger; trial fitting of the appropriate tray (upper or lower); preparation of the alginate with tap water at room temperature according to manufacturer’s instructions; insertion of the material into the tray and impression taking of the corresponding dental arch; careful removal of the tray after setting time; removal of the finger oximeter; and verification of correct data transfer and storage and documentation of notes in the *ViHealth* application.

#### 2.4.4. Digital Technique (Intraoral Scanner)

Intraoral scanning of the dental arches was performed using the Primescan 3D^®^ intraoral scanner (Dentsply Sirona Inc., Charlotte, NC, USA), strictly following the manufacturer’s instructions for acquiring high-precision digital images. The procedure was conducted with the participant seated, with the chair positioned between 45° and 60°, optimizing participant comfort and operator access. Prior to the procedure, the participant was informed about the intraoral scanner impression technique. HR and SpO_2_ were continuously monitored using the pediatric oximeter during the procedure, and data were stored in the *ViHealth* application.

The procedural steps included: pre-procedure explanation of the intraoral scanning process; placement of the oximeter on the participant’s right index finger; positioning of the scanner and initiation of the digital impression of the corresponding dental arch; completion of the scanning and verification of image quality in the scanner software; removal of the finger oximeter; and verification of correct data transfer and storage and documentation of notes in the *ViHealth* application.

#### 2.4.5. Interval Phase

An interval phase was implemented between the two impression procedures—an analog (alginate) and a digital (intraoral scanner) one—to allow HR and SpO_2_ to return to baseline levels. During this approximately 5 min interval, participants remained at rest, seated comfortably in a controlled environment, and free from external stimuli. Parents or legal guardians were allowed to be present, and participants were encouraged to engage in relaxing activities, such as talking or drawing, similarly to the initial rest phase.

Although the duration of the procedure varied depending on each child’s level of cooperation, the average time for both alginate and digital impressions was approximately 3 min. This comparable chair time suggests that the impression technique itself, rather than the duration, may have had a greater influence on anxiety levels

### 2.5. Statistical Analysis

Two outcomes were analyzed: the increment in heart rate (INCR HR) and the increment in oxygen saturation (INCR SpO_2_). These measures reflect the maximum variation occurring during each procedure and were calculated based on the difference between the extreme values recorded for each technique (alginate and intraoral scanner) from the beginning to the end of the procedure. The increment was determined using the following formula: INCR = Final Value − Initial Value.

Given their tendency toward a normal distribution, mean values and standard deviations were calculated. The normality of the distributions was assessed using the *Shapiro–Wilk* test. Since the assumption of normality was violated, the Friedman test (for repeated measures) was applied, which is appropriate for paired, non-parametric samples. When significant differences were identified among the groups, multiple comparisons for two paired samples were performed using the Wilcoxon signed-rank test with Bonferroni correction applied. The significance level was set at 5%.

For the statistical analysis, the software SPSS^®^ (IBM^®^ Corp., Released 2024. IBM^®^ SPSS^®^ Statistics for Windows^®^, Version 30.0. Armonk, NY, USA: IBM Corp.) was used.

## 3. Results

### 3.1. Graphical Analysis of Physiological Responses

**Figure 1:**
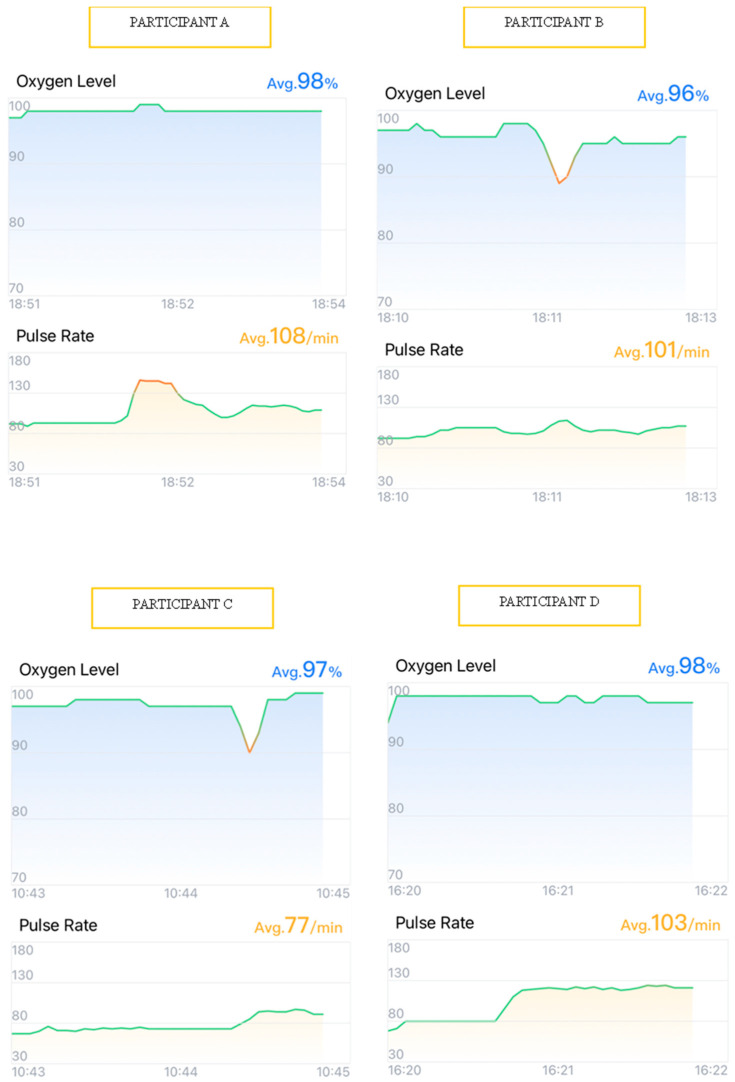
Graphs showing heart rate (HR) and oxygen saturation (SpO_2_) variation in 4 participants (A, B, C, and D) during the alginate impression technique.

**Figure 2:**
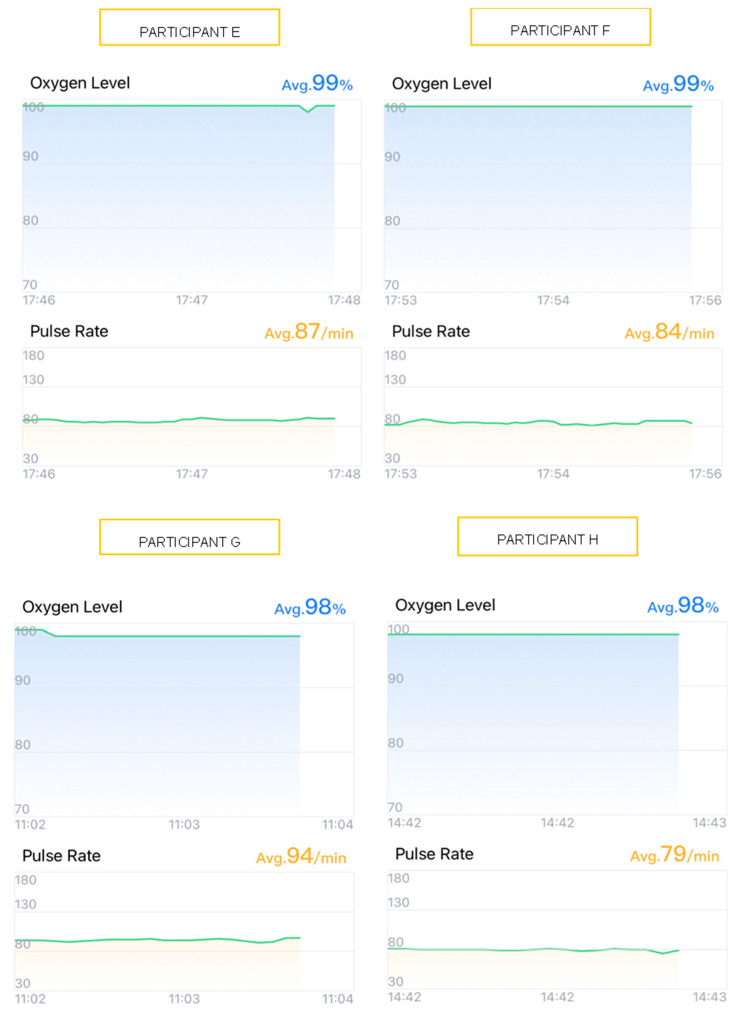
Graphs showing heart rate (HR) and oxygen saturation (SpO_2_) variation in 4 participants (E, F, G, and H) during the scanner impression technique. Source 2. Mobile application: *ViHealth*.

To illustrate the participants’ physiological responses, graphical data from four participants undergoing alginate impressions (Participants A, B, C, and D) (Figure 1) and four undergoing intraoral scanner impressions (Participants E, F, G, and H) (Figure 2) were selected. Visual analysis of heart rate (HR) and oxygen saturation (SpO_2_) variations, obtained via the *ViHealth* application, provided an enhanced understanding of the observed outcomes. These cases were selected as they reflect the general trends observed across all participants, exemplifying the typical patterns during both impression techniques. The graphs depict real-time registered HR and SpO_2_ fluctuations throughout the procedures.

Participants A–D, when subjected to alginate impressions, exhibited marked variations in both HR and SpO_2_ (Figure 1). For example, Participant A experienced an HR increase of nearly 50 beats per minute (bpm) during the insertion of the alginate tray into the oral cavity, while Participants B and C demonstrated significant SpO_2_ reductions to below 90%, indicative of transient hypoxemia episodes, accompanied by abrupt HR oscillations (Figure 1).

Conversely, participants E–H, when undergoing intraoral scanner impressions, displayed more stable tracings, with HR and SpO_2_ values remaining within normal ranges without significant fluctuations (Figure 2).

### 3.2. Descriptive Analysis

#### 3.2.1. Heart Rate (HR)

On average, HR increases were significantly higher during alginate impression making for both the maxillary and mandibular arches [(19 ± 11) bpm and (18 ± 9) bpm, respectively] compared to those during intraoral scanner impression making [(7 ± 5) bpm and (7 ± 4) bpm, respectively] (Table 1).

**Figure 3:**
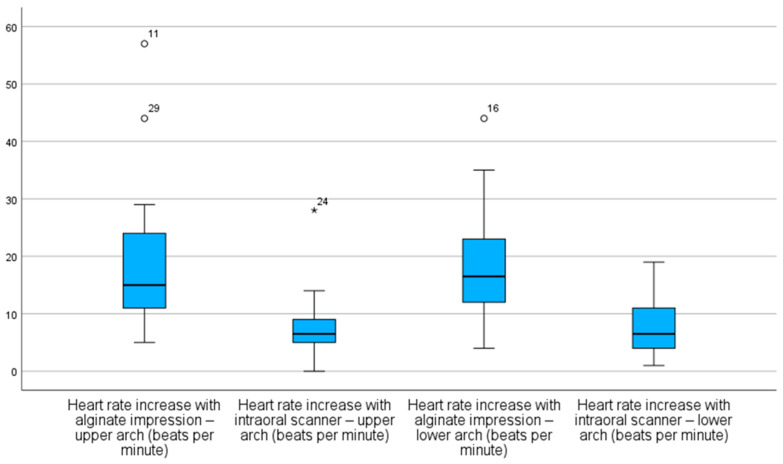
Distribution of heart rate (HR) increments across the different impression techniques. * value furthest from the other values.

On the other hand, scanner groups exhibited lower median values and more concentrated distributions, with minimal or no extreme outliers (Figure 3).

#### 3.2.2. Oxygen Saturation (SpO_2_)

Similar results were found for the SpO_2_ increment, with decreases being more pronounced when making alginate impressions for both arches [(−2 ± 2)% and (−2 ± 3)%, respectively] than when making scanner impressions [(−1 ± 1)% for both arches] (Table 2).

**Figure 4:**
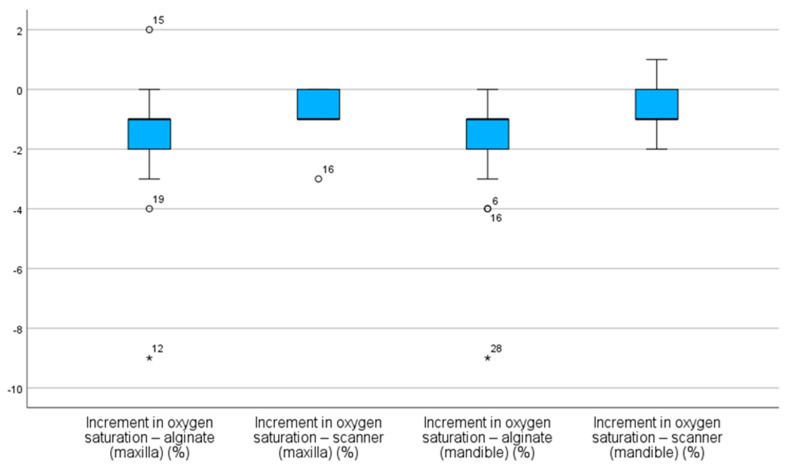
Distribution of oxygen saturation (SpO_2_) increments across the impression methods. * value furthest from the other values.

As observed, alginate impressions resulted in more significant SpO_2_ decreases in both arches when compared to intraoral scanner impressions. As shown in Figure 4 in both types of alginate impressions, the median of SpO_2_ increment was negative, indicating a decrease in SpO_2_ value during the procedure. This reduction is more pronounced when considering minimum values and outliers, particularly in the mandibular alginate group, where an extreme case showed a drop exceeding 8% (Figure 4).

### 3.3. InferentialAnalysis

#### 3.3.1. Heart Rate (HR)

Regarding the HR increment, statistically significant differences were observed between both impression methods, as evidenced by the Friedman test (χ^2^ = 49.30; *p* < 0.001).

Wilcoxon signed-rank tests (for paired samples) corroborated these findings, revealing statistically significant differences across all pairwise comparisons between the alginate and intraoral scanner techniques (*p* = 0.004), with consistently higher HR values recorded during alginate impression making (Table 3).

#### 3.3.2. Oxygen Saturation (SpO_2_)

Regarding the SpO_2_ increment, significant differences were also found between the impression techniques using the Friedemann test (χ^2^ = 21.41; *p* < 0.001). Regarding SpO_2_, statistically significant differences were found across all multiple comparisons (Table 4).

## 4. Discussion

Dental anxiety poses a significant challenge in pediatric dentistry, often hindering the delivery of appropriate treatments [2,13,23]. Understanding the factors and techniques that trigger this emotional state is crucial for pediatric dentists to develop treatment plans tailored to children’s emotional needs [13,33]. HR and SpO_2_ are physiological measures that are indicative of anxiety in children and have been used as reliable indicators of anxiety levels in previous studies [1,34,35,36].

This study aimed to analyze the effects of two dental impression methods—an analog (alginate) and a digital (intraoral scanner) one—on children’s anxiety levels by evaluating HR and SpO_2_ variations. The results indicated that alginate impressions led to increased HR and decreased SpO_2_, suggesting heightened anxiety among participants. These findings align with the ones presented in the study by Gandhi et al., who reported that children’s anxiety, reflected in HR and SpO_2_ levels, persisted despite the use of distraction techniques such as virtual reality and auditory stimuli [23].

In our study, the anxiety pattern was exhibited through the higher mean and median values observed in alginate groups, despite the presence of wider interquartile ranges, reflecting increased variability in physiological responses and potential influence from unassessed factors. Supporting these findings is the existence of extreme outliers, including cases where HR increases exceeded 40 bpm, indicating episodes of significant stress or discomfort during the procedure. In contrast, digital impressions with intraoral scanners resulted in more stable HR and SpO_2_ values, with distributions concentrated around the medians and with less pronounced outliers, suggesting a more controlled cardiovascular response and greater physiological comfort. Unlike conventional impressions, scanner impressions showed median SpO_2_ deviations closer to zero, indicating greater stability. The reduced dispersion and fewer negative outliers in these groups reinforce the notion that intraoral scanners are less invasive and more reliable procedures. Although this study did not directly assess scanner reliability, previous research has reported mixed findings on this aspect [37,38]. The study’s findings confirmed the initial hypotheses: higher anxiety levels, represented by increased HR and decreased SpO_2_, would be associated with conventional alginate impressions; intraoral scanner impressions would result in greater stability of these physiological parameters; and digital impression would be, from a physiological standpoint, the less anxiety-inducing technique.

Several studies have identified various factors contributing to anxiety in pediatric dentistry, including previous negative experiences, the use of needles, dental instrument noise, and the lack of distraction techniques [2,4,13,16,39,40,41,42]. Research comparing the two impression methods in children often relies on subjective measures like anxiety scales, patient reports, and visual questionnaires to assess children’s preference and perceived anxiety [22,25,26,28,29,31,32,43]. These assessment methods entail a degree of subjectivity when interpreting the results, especially in pediatric patients [44]. HR and SpO_2_ have been utilized in other studies to assess anxiety related to distraction techniques, local anesthesia, nitrous oxide sedation, benzodiazepine sedation, and various dental procedures [1,7,9,11,44,45,46,47]. This study stands out by employing physiological measures to evaluate the stress induced by impression techniques, potentially offering greater validity in pediatric populations.

The findings suggest that alginate impressions, by eliciting significant biological responses, are more invasive and associated with higher stress and anxiety levels in pediatric patients. The more favorable physiological response to intraoral scanners may be attributed to their less invasive nature, faster procedure times, and the lower likelihood of causing gag reflexes or discomfort [28]. Although previous studies [22,25,26,28,29,31,32] did not use objective physiological indicators, their conclusions, based on subjective reports, indicate greater discomfort associated with alginate impressions, often described as causing gagging and choking sensations and nausea. Therefore, despite methodological differences, the existing research supports the notion that digital impressions are more comfortable and less anxiety inducing for pediatric patients. Most studies concur that digital impression methods are preferred by children over traditional methods [22,25,26,28,29,31,32].

Although digital scanners involve a higher initial investment compared to conventional impression materials, they may prove cost-effective over time by reducing the need for repeated impressions, minimizing material waste, and shortening chairside time. These benefits not only improve workflow efficiency but also enhance the patient experience, particularly in pediatric settings where anxiety and cooperation can be challenging. As digital technology becomes more accessible and widely adopted, its integration into general pediatric dental practices is increasingly feasible and represents a practical alternative to traditional techniques.

This study has limitations, including the sampling method and sample size, which may restrict the generalizability of the findings to the population of children aged 5 to 11 years. Additionally, the sample was not paired, which may affect direct comparisons between techniques.

Previous experiences with impression techniques were not considered. Additionally, only one scanner with a universal tip was used; employing a scanner with a pediatric-specific tip might yield even more favorable results.

Another potential limitation is the influence of the operator’s behavior and the clinical setting itself, which may have affected the children’s responses. Factors such as the clinician’s tone, attitude, and familiarity with pediatric patients, as well as the overall environment, could play a role in modulating anxiety levels independently of the impression technique used.

Despite these limitations, the study’s results can inform future research and aid in developing more effective strategies to reduce fear and anxiety in children, fostering healthier relationships between dentists, children, and their caregivers. Moreover, comparing both alginate impressions and intraoral scanning in the same clinical context provides valuable insight into their performance for pediatric dental impressions.

Future studies could explore the impact of this methodology across different age groups, genders, specific systemic conditions (e.g., autism spectrum disorder), or prior dental experiences. Furthermore, longitudinal studies assessing patient responses over time or comparing various types of intraoral scanners would also be valuable.

## 5. Conclusions

Alginate dental impressions induced anxiety responses, as evidenced by increased HRs and decreased SpO_2_ levels. Intraoral scanner applications proved to be less invasive, resulting in milder variations in HR and SpO_2_. Intraoral scanners may serve as valuable tools in pediatric dentistry by reducing procedural distress for children, which is particularly beneficial for more anxious and less cooperative patients.

## Figures and Tables

**Table 1:** Heart rate increment (INCR (bpm)) according to impression method.

Impression Method and Arch Type	N	INCR (bpm) (MEAN ± SD)	CI _(0.95)_
Maxillary Arch—Alginate	30	19 ± 11	15–23
Maxillary Arch—Scanner	30	7 ± 5	5–9
Mandibular Arch—Alginate	30	18 ± 9	15–22
Mandibular Arch–Scanner	30	7 ± 4	6–9

INCR: Heart rate increment; bpm: Beats per minute; SD: Standard deviation; N: Sample size; CI: Confidence interval.

**Table 2:** Oxygen saturation increment (INCR (%)) according to impression method.

Impression Method and Arch Type	N	INCR (%) (MEAN ± SD)	CI _(0.95)_
Maxillary Arch—Alginate	30	−2 ± 2	−2–(−1)
Maxillary Arch—Scanner	30	−1 ± 1	−1–(−1)
Mandibular Arch—Alginate	30	−2 ± 3	−2–(−1)
Mandibular Arch—Scanner	30	−1 ± 1	−1–0

INCR: Oxygen saturation increment; SD: Standard deviation; N: Sample size; CI: Confidence interval.

**Table 3:** Multiple comparisons of heart rate increase (HR INCR) between impression methods.

Impression Method and Arch Type	HR INCR Maxillary Arch (bpm) Alginate	HR INCR Maxillary Arch (bpm) Scanner	HR INCR Mandibular Arch (bpm) Alginate	HR INCR Mandibular Arch (bpm) Scanner
HR INCR Maxillary Arch (bpm) Alginate	-	*p* = 0.004	-	-
HR INCR Maxillary Arch Scanner	-	-	*p =* 0.004	-
HR INCR Mandibular Arch (bpm) Alginate	-	-	-	*p* = 0.004
HR INCR Mandibular Arch (bpm) Scanner	*p* = 0.004	-	-	-

INCR: heart rate increase; bpm: beats per minute.

**Table 4:** Multiple comparisons of oxygen saturation increase (SpO2 INCR) between impression methods.

Impression Method and Arch Type	SpO_2_ INCR Maxillary Arch (%) Alginate	SpO_2_ INCR Maxillary Arch (%) Scanner	SpO_2_ INCR Mandibular Arch (%) Alginate	SpO_2_ INCR Mandibular Arch (%) Scanner
SpO_2_ INCR Maxillary Arch (%) Alginate	-	*p* = 0.056	-	-
SpO_2_ INCR Maxillary Arch (%) Scanner	-	-	*p =* 0.004	-
SpO_2_ INCR Mandibular Arch (%) Alginate	-	-	-	*p* = 0.004
SpO_2_ INCR Mandibular Arch (%) Scanner	*p* = 0.016	-	-	-

INCR: oxygen saturation increment; bpm: beats per minute.

## Data Availability

Research data are available upon request.

## Abbreviations

The following abbreviations are used in this manuscript:

HR	Heart rate
SpO_2_	Oxygen saturation
bpm	Beats per minute
FC INCR	Heart rate saturation increment
INCR SpO_2_	Oxygen saturation increment
